# Metabolic Disturbances in Adult-Onset Still’s Disease Evaluated Using Liquid Chromatography/Mass Spectrometry-Based Metabolomic Analysis

**DOI:** 10.1371/journal.pone.0168147

**Published:** 2016-12-22

**Authors:** Der-Yuan Chen, Yi-Ming Chen, Han-Ju Chien, Chi-Chen Lin, Chia-Wei Hsieh, Hsin-Hua Chen, Wei-Ting Hung, Chien-Chen Lai

**Affiliations:** 1 Faculty of Medicine, National Yang-Ming University, Taipei, Taiwan; 2 Division of Allergy, Immunology and Rheumatology, Taichung Veterans General Hospital, Taichung, Taiwan; 3 Ph.D. Program in Translational Medicine and Rong Hsing Research Center for Translational Medicine, National Chung Hsing University, Taichung, Taiwan; 4 Institute of Biochemistry, Microbiology and Immunology, Chung-Shan Medical University, Taichung, Taiwan; 5 Institute of Molecular Biology, National Chung-Hsing University, Taichung, Taiwan; National Research Council of Italy, ITALY

## Abstract

**Objective:**

Liquid chromatography/mass spectrometry (LC/MS)-based comprehensive analysis of metabolic profiles with metabolomics approach has potential diagnostic and predictive implications. However, no metabolomics data have been reported in adult-onset Still’s disease (AOSD). This study investigated the metabolomic profiles in AOSD patients and examined their association with clinical characteristics and disease outcome.

**Methods:**

Serum metabolite profiles were determined on 32 AOSD patients and 30 healthy controls (HC) using ultra-performance liquid chromatography (UPLC)/MS analysis, and the differentially expressed metabolites were quantified using multiple reactions monitoring (MRM)/MS analysis in 44 patients and 42 HC. Pure standards were utilized to confirm the presence of the differentially expressed metabolites.

**Results:**

Eighteen differentially expressed metabolites were identified in AOSD patents using LC/MS-based analysis, of which 13 metabolites were validated by MRM/MS analysis. Among them, serum levels of lysoPC(18:2), urocanic acid and indole were significantly lower, and L-phenylalanine levels were significantly higher in AOSD patients compared with HC. Moreover, serum levels of lysoPC(18:2), PhePhe, uridine, taurine, L-threonine, and (R)-3-Hydroxy-hexadecanoic acid were significantly correlated with disease activity scores (all p<0.05) in AOSD patients. A different clustering of metabolites was associated with a different disease outcome, with significantly lower levels of isovalerylsarcosine observed in patients with chronic articular pattern (median, 77.0AU/ml) compared with monocyclic (341.5AU/ml, p<0.01) or polycyclic systemic pattern (168.0AU/ml, p<0.05).

**Conclusion:**

Thirteen differentially expressed metabolites identified and validated in AOSD patients were shown to be involved in five metabolic pathways. Significant associations of metabolic profiles with disease activity and outcome of AOSD suggest their involvement in AOSD pathogenesis.

## Introduction

Adult onset Still's disease (AOSD), a systemic inflammatory disease, is characterized by fever, skin rash, arthritis, hepatosplenomegaly, variable multisystemic involvement, liver dysfunction, hyperferritinemia, and increase in acute phase reactants [1–2]. Although AOSD pathogenesis is not completely understood, it is thought to involve dysregulated immune response, cytokine-mediated inflammation, interaction between host and environmental factors, and genetic complexity [3–10].

Metabolomics is a novel and quantitative method of analyzing metabolite changes in a cell, tissue, or body fluids [11–14]. Metabolomics can be used to investigate overall metabolic profiles, taking into account genetic and environmental factors, and allowing for the integration of the effects of these factors [15]. Among the analytical methods used in metabolomics research, liquid chromatography/mass spectrometry (LC/MS) has been shown to be one of the best techniques in terms of selectivity, sensitivity, and reproducibility [16–17]. Multivariate statistical analysis, unsupervised principal component analysis (PCA) and supervised partial least squares-discriminant analysis (PLS-DA) are the most widely utilized methods for stratifying the different metabolic profiles between a patient group and a control group [18]. Given the integrated nature of systemic metabolism, the analysis of multiple metabolites may provide a better understanding of disease-associated metabolic changes, and further elucidate the involvement of biological pathways.

Accumulating evidence suggests that the metabolite profile is altered in patients with rheumatic diseases such as systemic lupus erythematosus (SLE) and rheumatoid arthritis (RA), and may be associated with disease activity [13,15,19–23]. The identification of metabolomic profiles in rheumatic diseases has provided insights into disease mechanisms, and highlighted their potential as biomarkers for diagnosis or prognosis [21–23]. Thus, we hypothesized that metabolomics could be used to detect disease-associated metabolic profiles, and identify metabolic biomarkers which may facilitate the diagnosis or prediction of disease outcome in AOSD. However, there are no data on the use of metabolomics in AOSD patients.

Therefore, this prospective study aimed to 1) identify the differentially expressed metabolites in AOSD patients using liquid chromatography/mass spectrometry (LC/MS)-based analysis, 2) quantify the differentially expressed metabolites using multiple reactions monitoring (MRM)/MS analysis, and 3) examine the associations of the altered metabolites with clinical characteristics and disease outcome in AOSD patients.

## Patients and Methods

### Patients

In this prospective study, 32 AOSD patients fulfilling the Yamaguchi criteria [24] were enrolled consecutively in the first stage of metabolomics analysis. In the replication analysis, we enrolled other 12 AOSD cases from another independent cohort for validation of the differentially expressed metabolites derived from the first stage of metabolomics. Disease activity of AOSD was assessed with a modified Pouchot score described by Rau et al. [25]. Based on the proposed classification of disease courses of AOSD [26–27], we had follow-up of the AOSD patients for at least one year, and classified them into three kinds of courses: monocyclic systemic course (only one episode of systemic manifestations, followed by complete remission within one year after disease onset); polycyclic systemic course (more than one episode of systemic manifestations, followed by partial or complete remission after onset of the initial or the subsequent attack); and chronic articular course (persistent arthritis involving above one joint and lasting longer than 6 months). We recruited 40 healthy volunteers (30 for the first stage of analysis and 12 for the replication analysis) without history of rheumatic disease as a control group. The ethics committee of the Institutional Review Board of Taichung Veterans General Hospital had approved this study (CE13320), and the written consent was obtained according to the Declaration of Helsinki.

### Serum samples preparation

**Fig 1:**
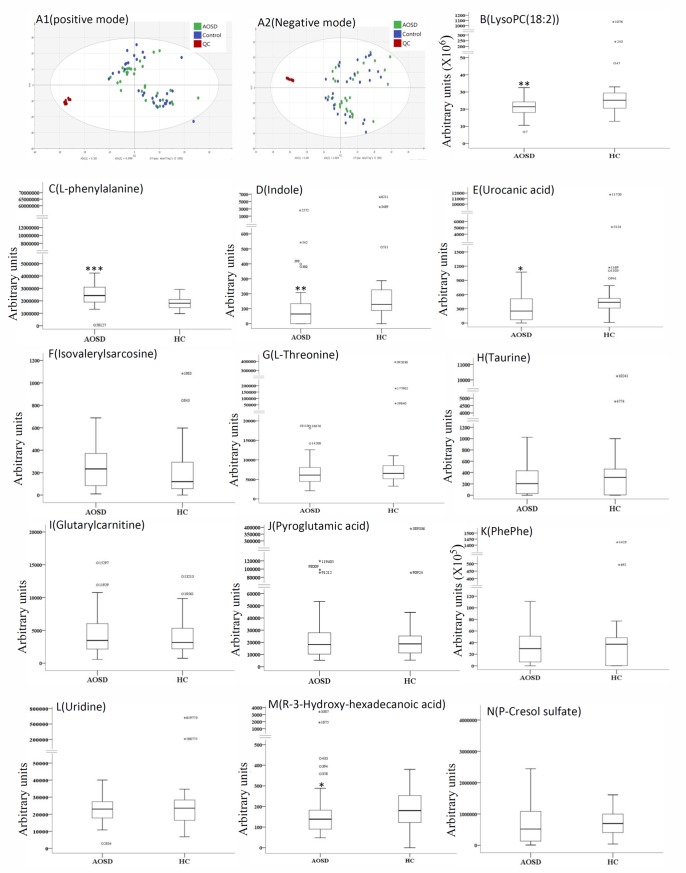
**A different clustering of metabolic ions was shown in the supervised orthogonal partial least squares-discriminant analysis (OPLS-DA) score plot (A1 and A2) from 32 patients with adult-onset Still’s disease (AOSD), 30 healthy subjects (control group), and quality control (QC).** The comparisons in serum levels of the differentially expressed metabolites between AOSD patients and control group (B-N). For the box plots, the bottom and top of the boxes represent the 25^th^ and 75^th^ percentile, respectively. The top and bottom bars represent the entire stretch of the data points for the subjects, except the extreme points which are indicated with exact values of data. The hyphen within box indicates the median value. *p<0.05, **p<0.01, ***p<0.001, versus the control group.

Obtained from venous blood samples which were taken in the morning after an overnight fast for 12 hours, the serum samples were stored at -80℃ until used for simultaneous determination of metabolic profiles. Prior to LC/MS analysis, 200μL of serum sample was mixed with 800μL of acetonitrile (Merck, NJ, USA), 5μL (50ppm) of esculetin and tubercidin, followed by vigorous vortex mixing for 2 min, and extraction with the aid of ultrasonication for 1 min. The sample mixtures were allowed to stand for 20 min at -20℃and then were centrifuged at 12,000 rpm for 30 min at 4°C. The supernatants of mixtures were transferred to new eppendorfs, and dried out with a turbovap nitrogen evaporator (AnytimeLabTrader, LLC, Oceanside, California, USA). Next, 100μL of mixture of acetonitrile–water (5:95 in volume) was added to each dried serum extract, and subsequently centrifuged at 12,000 rpm for 15 min at 4°C. The supernatants were then subjected to LC/MS analysis [16–17]. A quality control (QC) sample was prepared by mixing serum samples from 5 AOSD patients and 5 healthy subjects, and handled following the aforementioned procedure. The QC samples (1μL) were used for monitoring the stability of LC/MS analysis, and were continuously analyzed 3 times after 10 serum samples to validate the reproducibility of this platform (Fig 1A1–1A2).

### Determination of metabolite profiles using LC/MS analysis

LC/quadrupole time-of-flight (QTOF)-MS (Maxis Impact, Bruker, MA, USA) equipped with an electrospray ionization (ESI) source was used for analysis of metabolite profile. LC/MS was operated in positive and negative ion mode by applying a voltage of 3,300 V for positive ESI (ESI^+^) and 2,500 V for negative ESI (ESI^−^). The temperature of the heated capillary in the ESI source was set at 200°C. Next, a collision energy (represented as a percentage of a maximum possible energy) sufficient to fragment the precursor ion was used to produce product ion spectra. In this metabolite analysis, data-dependent scanning was adopted; MS scan events were set as full mass scan, followed by MS/MS scans of the **3** highest-intensity precursor ions for collision-induced dissociation using 35eV. Other source parameters were set as follows: scan range for mass/charge ratio, 50–1000 m/z; drying gas flow, 7L/min; and pressure of nebulizer gas, 0.8 bars.

### Chromatography using a Surveyor LC system

Eight microliter of the prepared sample was injected into a Waters Atlantis T3 C_18_ column (3 μm, 2.1 mm x 150 mm), and the eluent of the ultra-performance liquid chromatography (UPLC) (3000RSLCnano system, Dionex, Thermoscientific, N.W., Washington, D.C., USA) was directed to the ESI source of the mass spectrometer. The mobile phases consisted of a combination of solution A (0.1% formic acid in water) and solution B (0.1% formic acid in 100% acetonitrile). While maintaining a constant flow rate of 0.3 mL/min, the metabolites were eluted using linear gradients of 5–95% of B buffer from 0 to 14 min, and held at 95% of B buffer up to 17 min, after which it was decreased linearly to 5% at 17.1 min and held at 5% for 20 min until injection of the next analytical sample.

### Data processing and pattern recognition

The experimental data of UPLC−QTOF-MS were processed by Profile Analysis (Bruker software). The intensity of fragment ion was normalized with respect to the total ion count to generate a data matrix consisting of the retention time, the *m/z* value, and the normalized peak area. The processed data were exported and further analyzed by unsupervised PCA using EZinfo 2.0 software (UpdateStar, Berlin, Germany) after unit variance (UV) scaling. To enhance the separation seen with the PCA, supervised partial least squares -discriminant analysis (PLS-DA) using the EZinfo Software 2.0 (UpdateStar, Berlin, Germany) and orthogonal projections to latent structures-discriminant analysis (OPLS-DA) using SIMCA 14, version 1 (Umetrics, NFI Co., Ltd.) was carried out. By focusing on the metabolites that contributed to the separation of AOSD patients and control group, multivariate models based on ParetoVariance (Par) scaling were established. Student’s t test with the critical p-value <0.05 was selected to validate the significance of the metabolites selected. The corresponding fold change shows how these selected differential metabolites varied between the AOSD patients and control group. A probability of less than 0.05 was considered significant. The diagnostic sensitivity, specificity, and the area under receiver-operating characteristic (ROC) curve (AUC) were determined using SIMCA 14, version 1 (Umetrics, NFI Co., Ltd.).

### Identification of the specific metabolites

The differential *m/z* values were selected using the criteria with both variable importance for projection (VIP) value more than 1.0 and p-value <0.05 in t-test. The identities of specific metabolites were confirmed by comparing the differential *m/z* values to commercially available reference standards. The specific metabolites were also identified at the Scripps Center for Metabolomics and Mass Spectrometry (METLIN) (http://metlin.scripps.edu/). The biochemical reactions associated with these identified metabolites were obtained from the Human Metabolome Database (HMDB) (http://www.hmdb.ca/), the Kyoto Encyclopedia of Genes and Genomes (KEGG) (http://www.genome.jp/kegg/), Massbank (http://www.massbank.jp/), and Chemspider (http://www.chemspider.com/).

### Validation and quantification of the differentially expressed metabolites using MRM/MS analysis

UPLC analysis was performed on a Waters ACQUITY ultra performance LC system (Waters Corp., Milford, MA, USA), equipped with a binary solvent delivery system, an auto-sampler. The chromatography was performed on a Waters Acquity UPLC Atlantis T3 Column (100Å, 3 μm, 2.1 mm X 100 mm). Binary mobile phases with pahse A of water containing 0.1% formic acid (v/v) and phase B of ACN containing 0.1% formic acid (v/v) were used. The UPLC eluting conditions were optimized as follows: 0 min, 5% B; 14 min, 95% B; 17 min, 95% B; 17.1 min, 5% B; 20 min, 5% B. The total elapsed time required for a given chromatographic analysis was 20 min. The flow rate was 0.3 mL min^−1^. The column and the auto-sampler were maintained at 35°C and 16°C, respectively, and the injection volume of sample was 8 μL.

Mass spectrometry was operated on a Waters Xevo TQ tandem quadrupole mass spectrometer (Micromass MS Technologies, Manchester, UK) using an ESI operated in both positive and negative ion mode. The desolvation gas was set to 800 L h^−1^ at temperature of 400°C, and the source temperature was set to 150°C. The capillary voltage was set to 3.5 V, respectively. The collision gas flow was set at 0.21 mL min^−1^. Detection was carried out in multiple reactions monitoring (MRM) mode. Fifteen MS/MS functions were performed in a single UPLC run, the details of parameters are shown in Table 1. Tubercidin and esculetin were used as internal standard of positive mode and negative mode, respectively.

**Table 1:** Retention time and LC/MRM-MS parameters for 13 standard compounds and 2 internal standard.

Compound Name	Retention time tR (min)	Ion mode	Parent ion (m/z)	Daughter ion (m/z)	Dwell time (s)	Collision energy (V)	Cone voltage (V)
LysoPC(18:2)	11.81	Positive	520.54	104	0.02	28	38
LysoPC(18:2)	11.81	Positive	520.54	184.03	0.02	28	38
Indole	7.69	Positive	117.99	90.88	0.328	20	38
L-threonine	0.96	Positive	119.9	55.9	0.031	14	16
L-threonine	0.96	Positive	119.9	83.86	0.031	12	14
Taurine	5.35	Positive	125.8	98.3	0.019	8	26
Pyroglutamic acid	1.45	Positive	129.9	55.8	0.031	22	22
Pyroglutamic acid	1.45	Positive	129.9	83.85	0.031	32	22
Urocanoic acid	5.35	Positive	138.95	65	0.019	26	16
Urocanoic acid	5.35	Positive	138.95	92.84	0.019	64	16
L-phenylalanine	2.82	Positive	165.9	102.91	0.151	26	18
L-phenylalanine	2.82	Positive	165.9	130.91	0.151	14	18
Isovalerylsacosine	4.86	Positive	174.05	89.9	0.1	8	16
Isovalerylsacosine	4.86	Positive	174.05	84.89	0.1	12	16
Uridine	1.45	Positive	245	112.8	0.031	12	12
Glutarylcarnitine	1.77	Positive	276.2	198.94	0.031	16	28
Glutarylcarnitine	1.77	Positive	276.2	84.81	0.031	24	28
PhePhe	5.34	Positive	313.12	119.9	0.019	22	20
PhePhe	5.34	Positive	313.12	165.95	0.019	16	20
p-Cresol sulfate	5.84	Negative	186.98	106.89	0.086	22	28
p-Cresol sulfate	5.84	Negative	186.98	79.81	0.086	28	28
Hydropalmitic acid	14.49	Negative	271.2	58.8	0.5	22	28
Tubercidin	1.39	Positive	267.15	117.93	0.031	46	24
Tubercidin	1.39	Positive	267.15	134.9	0.031	20	24
Esculetin	4.75	Negative	177.04	104.87	0.005	20	36
Esculetin	4.75	Negative	177.04	148.83	0.005	28	34

### Mapping of AOSD-specific metabolic profiling and biological pathway

AOSD-specific metabolic profiling was mapped by comparing the identified metabolites to available reference standards and their biological pathways were analyzed using the MetaboAnalyst (http://www.metaboanalyst.ca/) tool.

### Statistical analysis

The results are presented as the mean ±standard deviation (SD) or median (interquartile range). The independent samples t test was used for between-group comparison of metabolites. Kruskal-Wallis test was used for between-group comparison of serum metabolites levels. When this test showed significant differences, the exact p-value was then determined using the Mann-Whitney U test. The correlation coefficient was obtained through the nonparametric Spearman’s rank correlation test. P values <0.05 were considered statistically significant.

## Results

### Clinical characteristics of AOSD patients

Among the 44 AOSD patients, 26 (59.1%) patients were in active status, which was defined as clinical activity score≧3. Among active patients, fever (≥39 ℃), rash, sore throat, arthritis, and lymphadenopathy were noted in 25 (96.2%), 19 (73.1%), 19 (73.1%), 14 (53.8%), and 12 (46.2%) patients respectively. There were no significant differences in the age at study entry (mean age ± standard deviation, 39.7±15.3 years versus 35.0±9.46 years), the proportion of females (33/44, 75.0% versus 33/42, 78.6%), serum creatinine levels, body mass index (BMI), or the proportion of past or present smoker between AOSD patients and healthy subjects.

### Serum metabolite profiles in AOSD patients and healthy controls

Based on UPLC−QTOF-MS detection and PCA analysis, marked differences in total ion chromatogram (TIC), in ESI^+^ mode and ESI^-^ mode, were observed between the patient group and the control group, with 907 positive ions and 1302 negative ions found. As illustrated in the OPLS-DA scores-plot, we observed different clustering of metabolites in the patient group and the control group in both ESI^+^ and ESI^-^ modes (Fig 1A1–1A2). After the screening of VIP (value above 1), 18 differentially expressed metabolites were detected in AOSD patents, as summarized in Table 2. Then, we validated 13 of these 18 differentially expressed metabolites using LC/MRM-MS analysis with pure standards, the transitions and MS parameters were shown in Table 1. Among these 13 metabolites, median levels of serum lysoPC(18:2), urocanic acid and indole were significantly lower, and levels of L-phenylalanine were significantly higher in AOSD patients compared with HC (Fig 1). There was no significant difference in serum levels of other metabolites between AOSD patients and the control group.

**Table 2:** Eighteen differentially expressed metabolites identified in AOSD relative to healthy control in the first stage of metabolomics analysis.

Ion-mode	No	tR (min)	m/z	Formula	Identified metabolites	AOSD versus Control				Related biological pathway
						Fold Change	*p-*value	VIP	AUC	
**ESI (+)**	1	12.27	520.34	C_26_H_50_NO_7_P	LysoPC(18:2)	1.52↓	**1.81E-02**	**6.41**	**0.606**	Phospholipid catabolism
	2	3.77	166.08	C_9_H_11_NO_2_	L-Phenylalanine	1.18↑	**5.52E-03**	**8.1**	**0.711**	Phenylalanine metabolism
	3	3.77	118.06	C_8_H_7_N	Indole	2.07↓	**1.78E-02**	**1.25**	**0.610**	Tryptophan metabolism
	4	10.27	139.05	C_6_H_6_N_2_O_2_	Urocanic acid	1.34↓	**2.68E-02**	**1.77**	**0.665**	Histidine metabolism
	5	3.27	174.11	C_8_H_15_NO_3_	Isovalerylsarcosine	1.47↑	**4.30E-02**	**1.23**	**0.641**	Glycine, serine, threonine metabolism
	6	5.77	120.06	C_4_H_9_NO_3_	L-Threonine	1.61↓	**2.71E-03**	**6.71**	**0.730**	Glycine and serine metabolism
	7	4.27	126.02	C_2_H_7_NO_3_S	Taurine	1.52↑	**5.10E-04**	**1.27**	**0.763**	Taurine and hypotaurine metabolism
	8	3.27	276.14	C_12_H_21_NO_6_	Glutarylcarnitine	1.57↑	**1.70E-02**	**1.24**	**0.634**	Fatty acid metabolism
	9	1.77	130.05	C_5_H_7_NO_3_	Pyroglutamic acid	1.23↑	**3.78E-02**	**1.34**	**0.616**	Glutathione metabolism
	10	5.77	313.15	C_18_H_20_N_2_O_3_	PhePhe	1.56↓	**7.98E-03**	**8.85**	**0.706**	Protein degradation and synthesis
	11	11.27	245.07	C_9_H_12_N_2_O_6_	Uridine	2.00↑	**9.00E-05**	**1.19**	**0.733**	Pyrimidine metabolism
	12	8.27	152.07	C_8_H_9_NO_2_	Dopamine quinone (DAQ)	4.74↓	**0.00E+00**	**1.3**	**0.765**	Tyrosine metabolism
**ESI (−)**	13	7.27	187	C_7_H_8_O_4_S	P-Cresol sulfate	1.78↓	**3.36E-02**	**10.1**	**0.641**	Gut microbial metabolism
	14	16.27	271.22	C_16_H_32_O_3_	(R)-3-Hydroxy-hexadecanoic acid	1.39↑	**3.85E-02**	**1.42**	**0.655**	Fatty acid biosynthesis
	15	11.27	367.15	C_15_H_29_O_8_P	PA(6:0/6:0)	2.18↓	**2.40E-04**	**14.8**	**0.791**	Fatty acid metabolism
	16	11.27	293.13	C_16_H_22_O_5_	Tocopheronic acid	2.39↑	**1.02E-03**	**6.73**	**0.861**	Fatty acid metabolism
	17	12.27	476.26	C_23_H_44_NO_7_P	LysoPE(18:2/0:0)	1.50↓	**7.50E-04**	**5.35**	**0.752**	Phospholipid catabolism
	18	4.27	206.07	C_8_H_17_NOS_2_	Dihydrolipoamide	1.99↓	**1.92E-03**	**1.40**	**0.705**	Valine, leucine, isoleucine metabolism

t_R_: retention time; ESI: electrospray ionization; VIP: variable importance in the projection; VIP values were calculated using OPLS-DA

↑ indicates up-regulated and ↓ indicates down-regulated. AUC: the area under receiver-operating characteristic curve.

Fold change was calculated as log_2_ (average peak intensity of patient group/average peak intensity of healthy control group).

Regarding the performance in disease diagnosis, the ROC curve analysis (the patient group versus the control group) revealed that the AUC reached 0.95 (p<0.01) with a high specificity (96.7%) and moderate sensitivity (78.1%) in the first stage of metabolomics, and the AUC reached 0.972 (p<0.001) with a high specificity (100%) and high sensitivity (91.67%) in the replication analysis.

### Association of metabolites with disease activity or clinical manifestations

**Fig 2:**
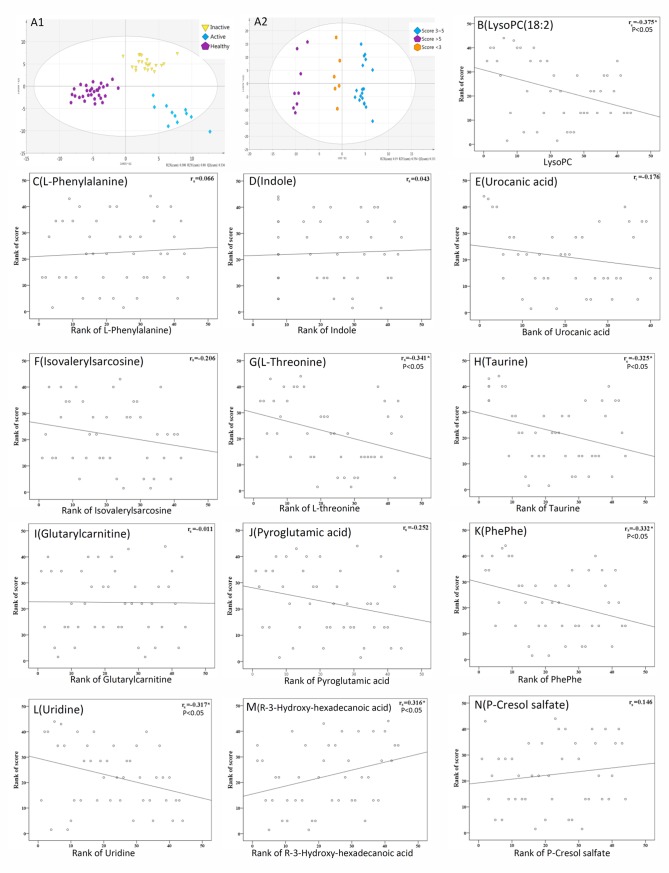
**A different clustering of metabolic ions was shown in the OPLS-DA score plot from 14 active AOSD patients, 18 inactive AOSD patients, and 30 healthy controls (A1).** A different clustering of metabolic ions was shown in AOSD patients with different activity scores (A2). The correlation between serum levels of the altered metabolites and AOSD activity scores (B-N).

As illustrated in the OPLS-DA scores-plot, different clustering of metabolic ions was noted among active AOSD patients, inactive AOSD patients, and the control group (Fig 2A1 and S1 Fig). Different clustering of metabolites was also observed in AOSD patients with different activity scores (Fig 2A2 and S2 Fig). Among the differentially expressed metabolite, serum levels of lysoPC(18:2), PhePhe, taurine, and L-threonine were negatively correlated, while levels of (R)-3Hydroxy-hexadecanoic acid were positively correlated with clinical activity scores in AOSD patients (Fig 2B–2N). However, there were no significant associations of the differentially expressed metabolite with clinical manifestations in AOSD patients.

### The association of metabolomic profiles with disease outcome in AOSD patients

**Fig 3:**
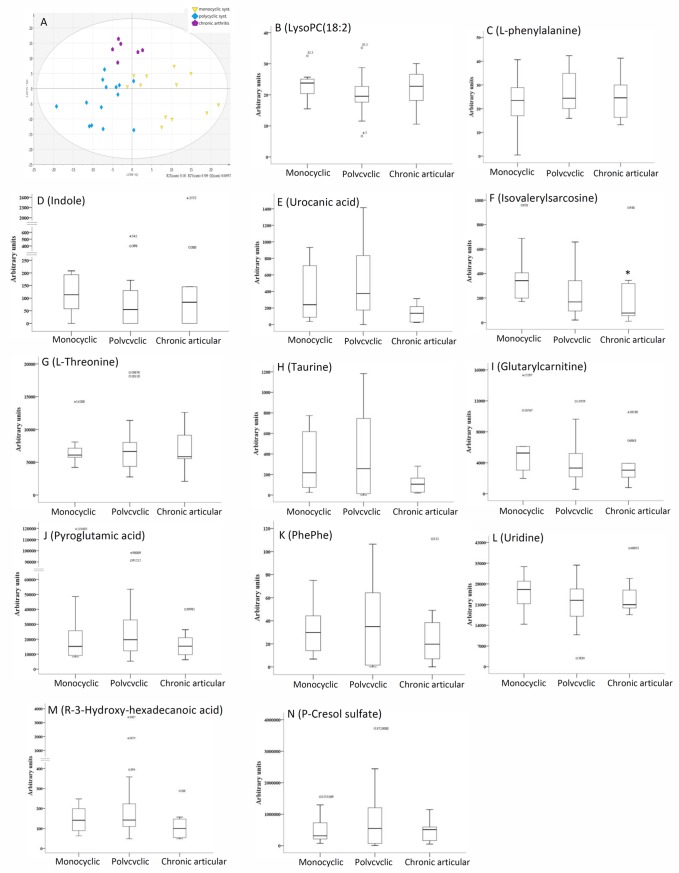
**A different clustering of metabolic ions in the OPLS-DA score plots from AOSD patients with different patterns of disease outcome (A).** The comparisons in serum levels of the differentially expressed metabolites in AOSD patients with different patterns of disease outcome (B-N). *p<0.05, versus monocyclic systemic pattern or polycyclic systemic pattern. The explanations for box plots s are as those described in Fig 1 legend.

As illustrated in the OPLS-DA scores-plot, AOSD patients with different patterns of disease outcome revealed different clustering of metabolites (Fig 3A). Moreover, significantly lower levels of isovalerylsarcosine were observed in AOSD patients with chronic articular pattern (median, 77.0AU/ml) compared with those with monocyclic systemic (341.5AU/ml, p<0.05) or polycyclic systemic pattern (168.0AU/ml, p<0.05) (Fig 3). There was no significant association of disease outcome with other metabolites.

### AOSD-specific metabolic mapping and biological pathway

**Fig 4:**
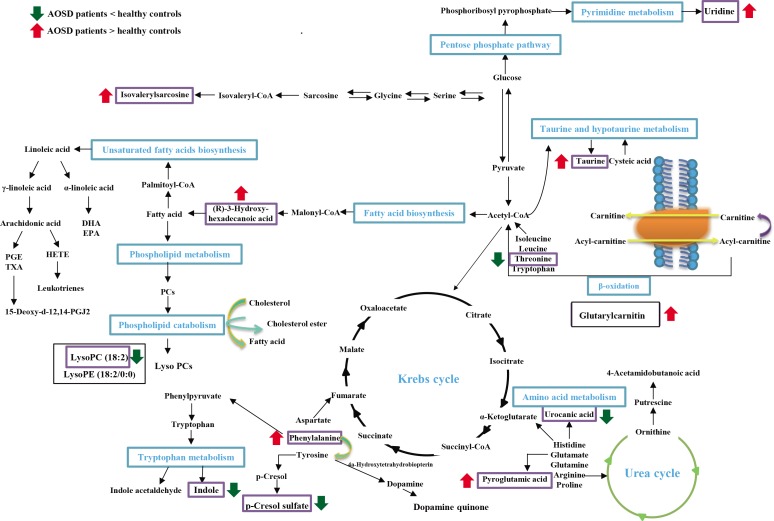
Schematic representation depicts the metabolic imbalance in patients with adult-onset Still’s disease (AOSD). (↑) represents up-regulated changes observed in AOSD; (↓) represents down-regulated changes observed in AOSD.

As illustrated in Fig 4, the most discriminative metabolites in AOSD were organized into different biochemical pathways, including amino acid metabolism, fatty acid biosynthesis, phospholipid metabolism, taurine metabolism, and pentose phosphate metabolism.

## Discussion

LC/MS-based metabolomics approach has potential implications for disease diagnosis and prediction [18,28–30]. Our study is the first to use UPLC-QTOF/MS-based metabolomics to reveal 18 differentially expressed metabolites in AOSD patients compared with healthy subjects, and then use LC/MRM-MS analysis to validate 13 metabolites in replication cohort with a high AUC and specificity. These significant metabolites, as shown in Fig 4, are potentially involved in the metabolism of amino acid, fatty acid, phospholipid, taurine, and pentose phosphate. Moreover, serum levels of lysoPC(18:2), PhePhe, uridine, taurine, L-threonine, and (R)-3-Hydroxy-hexadecanoic acid were significantly correlated with clinical activity scores (all p<0.05) in AOSD patients. Significantly lower levels of isovalerylsarcosine were also observed in patients with chronic articular pattern compared with other patterns of disease outcome in AOSD. These observations suggest these altered metabolites might be involved in AOSD pathogenesis.

Using standard compounds and LC/MRM-MS analysis, we revealed significantly lower levels of lysoPC(18:2), urocanic acid and indole in AOSD patients compared with the control group. LysoPC(18:2) is one of the class of phospholipids that are intermediates in the metabolism of phospholipids [31]. The abnormal metabolism of phospholipid may promote the systemic inflammatory state [32]. In our study, the decreased levels of lysoPC(18:2) in AOSD patients may indicate its potential protective effect aganst inflammatory response and suggest its augmented consumption in apopotic processes in this disease [33]. Urocanic acid is one metabolite which is involved histidine metabolism. The results of a recent study showed urocanic acid derivatives have anti-inflammatory effects on inflammatory bowel disease [34], supporting the decreased levels of urocanic acid in our AOSD patients. Indole, another important metabolite, is a product of amino acid (tryptophan) metabolism. We revealed the decreased indole levels in AOSD patients, which was similar to previous finding that tryptophan was down-regulated in patients with ankylosing spondylitis compared with controls [35], and suggested the depletion of tryptophan during inflammation may serve as a damping effect. Interestingly, p-Cresol sulfate (PCS), solely produced by the gut microbiota, can enhance the production of reactive oxygen species and then activate the nuclear factor-kappaB pathway, resulting in both oxidative stress and pro-inflammatory cytokine production [36]. Although the statistical significance was not reached (p = 0.095), decreased PCS levels in our patients might indicate the down-regulation of inflammatory and oxidative mechanisms in AOSD. These findings may link the altered microbial metabolites with the pathogenesis of rheumatic diseases [22]. In contrast, we revealed significantly higher levels of L-phenylalanine in patients compared with the control group. The results supported the findings of other previous studies showing that phenylalanine levels increased in chronic inflammation [37] and the urine phenylalanine levels increased in adjuvant-induced arthritis rats [38].

In this study, the different clustering of metabolites observed in AOSD patients with different activity scores indicated that the status of inflammation could be reflected in the metabolomic profiles [15]. As shown in Fig 2, serum levels of lysoPC(18:2), PhePhe, uridine, taurine, L-threonine, and (R)-3-Hydroxy-hexadecanoic acid were significantly correlated with activity scores (all p<0.05) in AOSD patients. Interestingly, we also demonstrated differential clustering of metabolites between inactive AOSD patients and healthy subjects, as shown in Fig 2A1, suggesting that the disease remission state in AOSD is not the result of total normalization of metabolic change. Longitudinal studies will be needed to monitor the change in metabolites before and after treatment in order to identify biomarkers helpful for optimizing therapy as in previous studies [23].

The disease course of AOSD patients may vary considerably [26–27]. OPLS-DA plot, as shown in Fig 3A, demonstrated different clustering of metabolites among different patterns of disease outcome in AOSD patients. Among the differentially expressed metabolites, serum levels of isovalerylsarcosine were significantly lower in AOSD patients with chronic articular pattern compared with other patterns of disease outcome. Isovalerylsarcosine, an important metabolite, is a product of glycine, serine and threonine metabolism. A decrease in serine and threonine content of the cartilage proteoglycan in antigen-induced arthritis of rabbit [39], supporting our findings showing the decreased levels of isovalerylsarcosine in AOSD patients with chronic articular pattern of disease outcome.

As illustrated in Fig 4, the multiple potential biochemical pathways involved in AOSD-related metabolomic profiling highlight the complexity of the metabolic response to systemic inflammation in this disease. Because the serum samples represent a cross-section of metabolic events reacting to multiple independent triggers, it is probable that subsets of these metabolic changes are interconnected, and/or associated with immune activation in AOSD.

This pilot study still had some limitations. The lack of significant associations of the differentially expressed metabolites with clinical features may be due to the small sample size in this clinically heterogeneous and rare disease: its prevalence estimated to be lower than 1 case per 100,000 people [40]. This study was cross-sectional in design and thus it is possible that the metabolic profiles could alter with disease progression or therapeutic strategies. In addition, we did not elucidate the biological function of these metabolites. Future studies dissecting the functional roles and pathogenic mechanisms of these significant metabolites are clearly needed.

## Conclusions

Our metabolomics based on both UPLC/TOF-MS and LC/MRM-MS analysis identified 13 differentially expressed metabolites which were associated with the metabolism of amino acid, fatty acid, phospholipid, taurine and pentose phosphate in AOSD. Significant associations of metabolic profiles with disease activity and outcome suggest their involvement in AOSD pathogenesis. The diagnostic or prognostic utility of these altered metabolites need to be evaluated by studies with a larger number of AOSD patients or of other phenotypically similar diseases [20,41].

## Supporting Information

S1 Fig The loading plot for the OPLS-DA score plot from 14 active AOSD patients, 18 inactive AOSD patients, and 30 healthy controls. (TIF)

S2 Fig The loading plot for the OPLS-DA modeling plot from AOSD patients with different disease activity scores. (TIF)

## Abbreviations

AOSD: Adult onset Still's disease
AUC: area under receiver-operating characteristic (ROC) curve
DAQ: dopamine quinone
ESI: electrospray ionization
LC/MS: liquid chromatography/mass spectrometry
MRM: multiple reactions monitoring
NF-kB: nuclear factor-kappaB
OPLS-DA: orthogonal projections to latent structures-discriminant analysis
PCA: principal component analysis
PC: phosphatidylcholine
PCS: p-Cresol sulfate
PE: phosphatidylethanolamine
PLS-DA: partial least squares-discriminant analysis
QC: quality control
QTOF: quadrupole time-of-flight
RA: rheumatoid arthritis
SLE: systemic lupus erythematosus
TIC: total ion chromatogram
UPLC: ultra-performance liquid chromatography
UV: unit variance
VIP: variable importance for projection
