# Supplementary figures and images for: Metabolic Disturbances in Adult-Onset Still’s Disease Evaluated Using Liquid Chromatography/Mass Spectrometry-Based Metabolomic Analysis
Source: PLoS One. 2016 Dec 22;11(12):e0168147. doi: 10.1371/journal.pone.0168147 (PMC5179000; doi:10.1371/journal.pone.0168147)

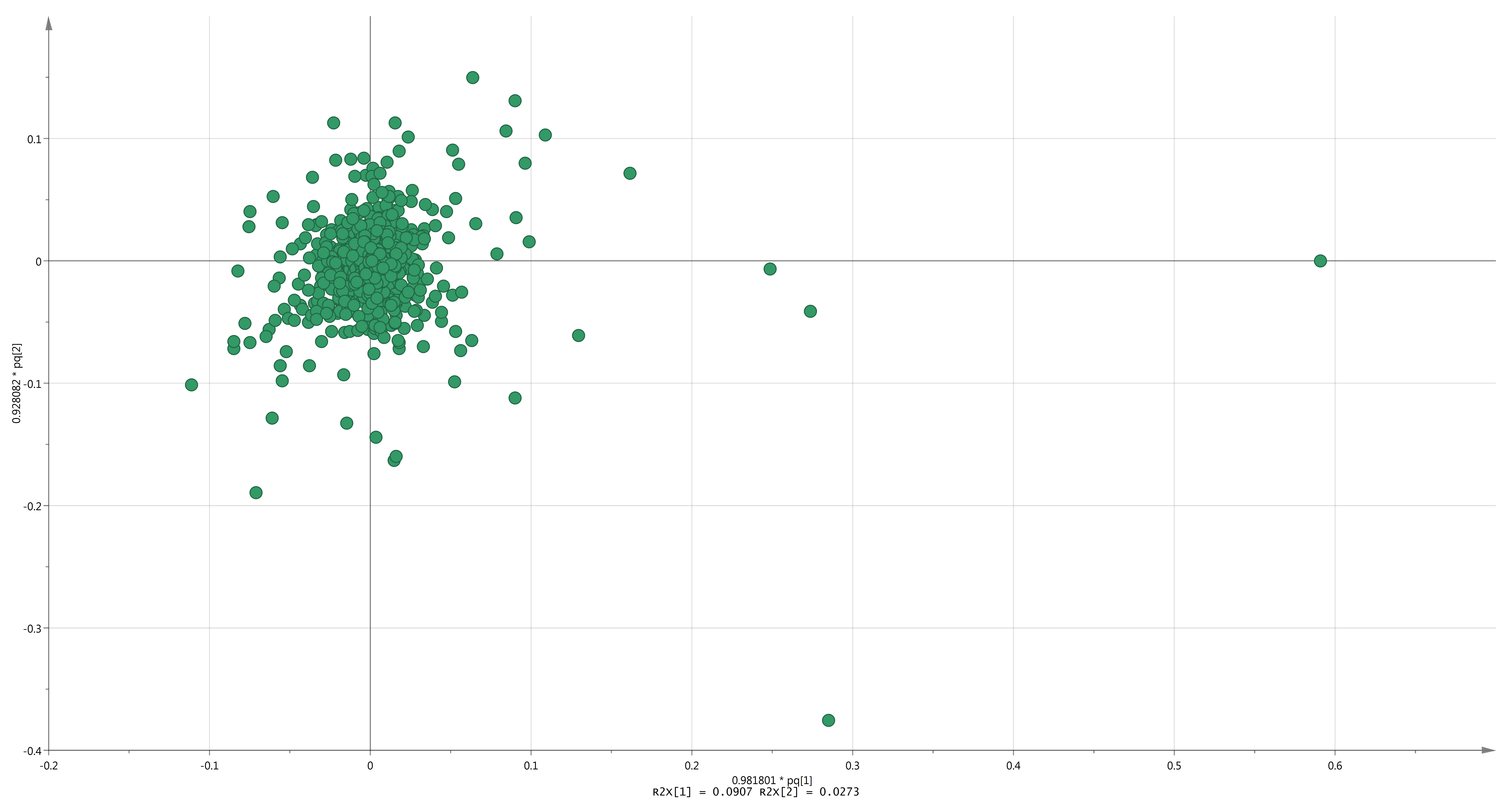

Supplement: S1 Fig — (TIF) [file pone.0168147.s001.tif]

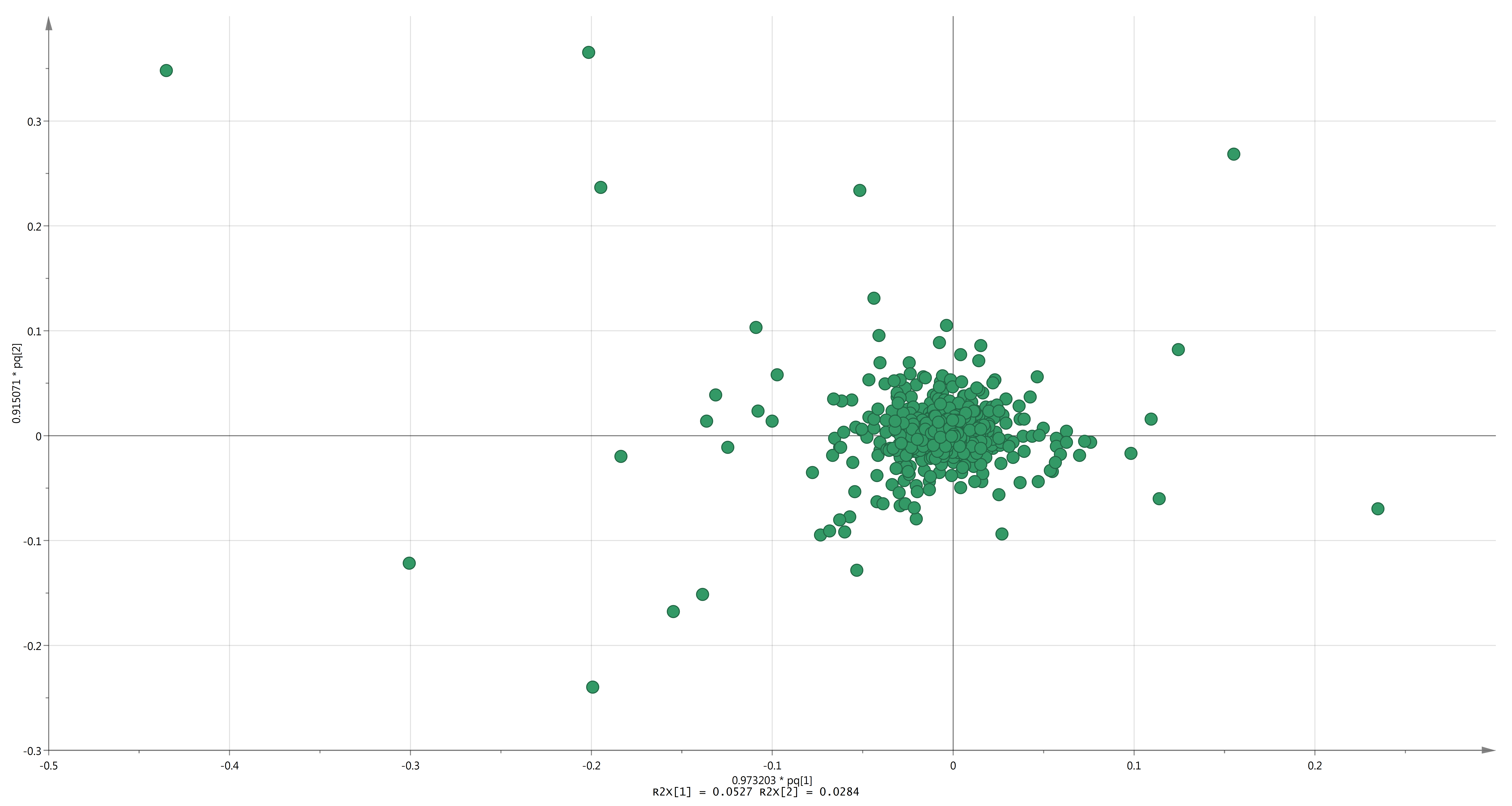

Supplement: S2 Fig — (TIF) [file pone.0168147.s002.tif]
